# Radiation induced changes in profibrotic markers in the submental muscles and their correlation with tongue movement

**DOI:** 10.1371/journal.pone.0287044

**Published:** 2023-06-23

**Authors:** Suzanne N. King, Nada Kaissieh, Chandler Haxton, Marjan Shojaei, Luke Malott, Lekha Devara, Rebecca Thompson, Kate L. Osman, Jessica Millward, Megan Blackburn, Teresa E. Lever

**Affiliations:** 1 Department of Otolaryngology–Head and Neck Surgery and Communicative Disorders, School of Medicine, University of Louisville, Louisville, KY, United States of America; 2 Department of Otolaryngology—Head and Neck Surgery, University of Missouri School of Medicine, Columbia, MO, United States of America; 3 Department of Radiation Oncology, School of Medicine, University of Louisville, Louisville, KY, United States of America; Wake Forest Baptist Medical Center, UNITED STATES

## Abstract

Swallowing impairment is a major complication of radiation treatment for oropharyngeal cancers. Developing targeted therapies that improve swallowing outcomes relies on an understanding of the mechanisms that influence motor function after radiation treatment. The purpose of this study was to determine whether there is a correlation between radiation induced changes in tongue movement and structural changes in irradiated submental muscles, as well as assess other possible causes for dysfunction. We hypothesized that a clinically relevant total radiation dose to the submental muscles would result in: a) quantifiable changes in tongue strength and displacement during drinking two months post treatment; and b) a profibrotic response and/or fiber type transition in the irradiated tissue. Sprague-Dawley adult male rats received radiation to the submental muscles at total dose-volumes known to provoke dysphagia in humans. A clinical linear accelerator administered 8 fractions of 8Gy for a total of 64Gy. Comparisons were made to sham-treated rats that received anesthesia only. Swallowing function was assessed using videofluoroscopy and tongue strength was analyzed via force lickometer. TGFβ1 expression was analyzed via ELISA. The amount of total collagen was analyzed by picrosirius red staining. Immunofluorescence was used to assess fiber type composition and size. Significant changes in licking function during drinking were observed at two months post treatment, including a slower lick rate and reduced tongue protrusion during licking. In the mylohyoid muscle, significant increases in TGFβ1 protein expression were found post radiation. Significant increases in the percentage of collagen content were observed in the irradiated geniohyoid muscle. No changes in fiber type expression were observed. Results indicate a profibrotic transition within the irradiated swallowing muscles that contributes to tongue dysfunction post-radiation treatment.

## Introduction

Radiation based treatments are known to compromise swallowing function in survivors of head and neck squamous cell carcinoma [1,2]. The resulting dysphagia leads to long-term alterations in diet consistencies, reduced nutritional intake, and reduced quality of life [3,4]. Clinical research has shown abnormal changes in swallowing kinematics occur early in the pathologic course of radiation-induced dysphagia, prior to the onset of clinical symptoms of dysphagia (i.e., aspiration, oropharyngeal residue) [5,6]. This was supported by animal studies demonstrating abnormal changes in bolus size, licking function, and tongue force present 1–2 weeks after radiation treatment ends [7,8]. Gene analysis of irradiated swallowing muscles has also revealed upregulation in growth factor genes around the same time post-treatment [9]. These findings suggest early adaptations in the irradiated muscles are associated with development of radiation-induced dysphagia. The current study aimed to assess possible mechanisms for post radiation changes to swallowing muscles that may be linked to functional changes in swallowing.

Deficits in tongue function can significantly contribute to problems in transporting the bolus from the oral cavity and through the pharynx during swallowing. Radiation to the rat submental muscles has shown that decreases in licking rate and increases in time between licks occurs starting around the last week of treatment [10], which suggests that tongue movement may be affected by radiation to this region. Previous work has demonstrated that radiation to the genioglossus or hyoglossus muscles, which are directly involved in lingual movement, leads to a reduction in maximum tongue muscle contraction and a decrease in tongue displacement in anesthetized animal models [8,11]. However, the tongue uses submaximal strength during swallowing [12]. Therefore, it is unclear from these studies if the decline in tongue strength causes changes in licking and swallowing post radiation. Given that these studies focused irradiation directly to the tongue, it is also unknown if irradiating other swallowing muscles results in changes in tongue function. Our previous work has shown that injuries to the mylohyoid muscle can impact the motor activity of other oropharyngeal structures during swallowing [13]. This is likely because of the sensorimotor integration involved in swallowing, since swallowing related cranial nerves (i.e., V, IX, X, and XII) have extensive anatomical and functional linkages via the brainstem [14–16]. Further work is warranted to determine if radiation of the submental muscles impairs tongue force and/or displacement during drinking behavior and if these dysfunctions correlate with structural changes to the irradiated muscles.

The literature on radiation classifies injuries based primarily on the tissue (mucosa versus muscle) being irradiated. Direct radiation damage to the underlying lamina propria, muscle, or nerve without involvement of the mucosa is referred to as generic or primary radiation injury. Previous studies have characterized primary radiation injuries as gradual changes in the tissue starting immediately post treatment that worsen over time, eventually resulting in significant fibrosis and dysfunction [17]. Muscle injuries that lead to disruptions in the extracellular matrix initially start with an upregulation in profibrotic factors, i.e., transforming growth factor (TGF)β1, which initiates a cascade of events leading to synthesis and/or degradation of collagen content [18]. Previous research has also shown post radiation changes relative to the incidence and size distribution of muscle fiber types at 2-weeks post treatment to the leg or laryngeal muscles, which could directly alter a muscle’s contractile and metabolic properties [19,20]. Further analysis is needed to determine if irradiated swallowing muscles transition to a profibrotic phenotype and/or alter their fiber type composition during early stages post radiation.

The underlying mechanism of radiation-induced dysphagia is unknown. Long-held clinical belief suggests that dysphagia is caused by fibrosis of the swallowing muscles. This notion has not been supported by animal studies, as muscle weakness in the tongue has been observed post-radiation without histological signs of fibrosis. However, the typical mean dose thresholds to the human submental muscles that provoke dysphagia have been reported to range between 45-70Gy [21–23], which is above the threshold utilized in previous studies. Because post radiation tissue changes are known to be dose-dependent [19], it is possible that clinically relevant dose volumes result in more significant tissue damage than previous animal models have depicted.

“Teasing out” the relative contribution of each cancer treatment (i.e., surgery, radiation therapy, chemotherapy, or a combination thereof), requires systematic investigations that are not feasible for clinical studies. We have therefore developed a rat model to facilitate the investigation of causative factors of dysphagia associated with radiation treatment [7–10]. Our radiation model specifically targets the submental muscles (anterior digastric, mylohyoid, and geniohyoid) that form the floor of the mouth, which spans from the mandible to the hyoid bone. Importantly, the submental muscles are essential to a variety of oral stage swallowing behaviors, such as opening the mouth to take a sip/bite, chewing, and bolus formation. They also are part of the “leading complex” of muscles recruited at the onset of the pharyngeal stage of swallowing [24]. Activation of the submental muscles (via the trigeminal nerve, CN V) during swallowing aids in posterior base of tongue movement [25] and elevates the hyoid and larynx in synchrony [26]. This results in epiglottic deflection to cover/protect the airway while also pulling open the upper esophageal sphincter to allow food/liquid to enter the esophagus without aspiration into the airway. The aim of the current study is to determine whether there is a correlation between tongue dysfunction and the structural changes following clinically relevant total doses of radiation to the submental muscles.

There were two objectives to this study. The first was to determine if impairments in tongue movement or strength are associated with aberrancies in lingual behaviors after radiation to the submental muscles. We hypothesized that targeted irradiation of the submental muscles in rats would result in quantifiable changes in tongue displacement and strength during drinking. The second objective was to identify structural adaptations that maybe driving aberrant swallowing function using a clinically relevant total radiation dose. We hypothesized that radiation would induce a profibrotic phenotype (i.e., increases in TGFβ1 and total collagen content) and alter fiber type composition in the irradiated submental muscles. We also hypothesized that these structural and molecular changes will correlate with the alterations in tongue displacement.

## Methods

All experimental protocols were approved by the Institutional Animal Care and Use Committees of the University of Louisville and the University of Missouri. Fourteen adult male Sprague-Dawley rats (12 months of age; 450-520g) underwent 64Gy of radiation or sham treatment. These animals were previously used in experiments to study behavior changes during the course of radiation treatment [10]. Rats were assigned to the radiation group if they were able to burrow. Burrowing is a rudimentary behavior in rats. Baseline performance indicates that the rodent can perform this behavior and does not signify activity/stress level; therefore, this method for assigning animals into the study groups does not bias other behavioral tests. Two weeks post treatment, the animals were transported to the University of Missouri for further swallow-related kinematic/strength analysis assessments. The animals spent three weeks acclimating to the new environment prior to starting training and testing. Data were collected 2 months post-radiation. The time point was chosen based on previous work showing declines in tongue muscle force presenting at 2-weeks post radiation and persisting up to 5-months post [11].

### Muscle selection

The small size of the submental muscles limits the number of assays that can be performed. To minimize the number of animals used for these experiments, we employed different submental muscles for each assay. The mylohyoid and geniohyoid muscles were our primary targets of radiation as they play a major role in swallowing and radiation to these muscles has been linked to dysphagia. The mylohyoid muscle was used for ELISA. The geniohyoid muscle was chosen for histology and immunofluorescence analysis based on feasibility, as obtaining cross sections of the mylohyoid muscle can be challenging due to the thin size of the muscle. The anterior digastric muscle was also included in histological analysis to allow for comparisons between irradiated muscles, since it lies within the radiation field.

### Radiation procedure

A clinically relevant radiation dose scheme was devised based on previous reports in humans showing a high incidence of dysphagia with total dose-volumes between 40-70Gy to the submental muscles [21–23]. The submental muscles in the rat were exposed to a total dose of 64Gy of radiation given in 8 fractions of 8Gy. This total dose-volume was employed as it is known to provoke dysphagia in humans [21–23]. One fraction was given per day and max of 3 fractions were administered per week. Rodents had an unplanned 2-week break between the 3^rd^ and 4^th^ fractionated doses due to a failure with the instrument. Since we are utilizing hypofractionated radiotherapy approach, the break between fractions could have induced greater skin irritations during treatment. To limit this irritation, all animals underwent a skin care regimen that included cleaning and treating with aloe vera gel. Sham-treated controls underwent anesthetization and immobilization for a similar duration and days per week as the radiated group, without radiation exposure.

Rodents were anesthetized with 2–4% of isoflurane. Radiation treatment was administered as previously described [7,9], employing a Clinac iX linear accelerator (Varian Medical Systems, Palo Alto, CA, USA) with 6MeV electrons using a 10cm applicator and a standard 4x4 cm^2^ insert to collimate the electron beam. Briefly, animals were positioned supine and immobilized on the treatment bed. A 0.5 cm layer of Superflab material (CNMC, Nashville, TN) was placed on the outer surface of the skin above the submental space to reduce the maximal dose penetration depth of the electron beam to the submental muscles (~1.25–1.75 cm in depth). The distance between the radiation source to the surface of the Superflab material was held at a constant 100 cm for each animal. A 0.3 cm lead shield was then placed anterior to the Superflab material to further collimate the beam to the dimensions of the submental space (~11x 10mm).

### Tongue Bead Injection

One month after radiation treatment, rats were anesthetized with isoflurane and radiopaque markers were implanted into the tongue to facilitate quantification of tongue displacement during VFSS testing. Tantalum bead fiducial markers (0.5 mm diameter; Bal-tec, CA) were injected into the tip and mid portion of the tongue with a 21 gauge, 1-inch needle and a custom-formed fine wire plunger. Bead placement was assessed using fluoroscopy and later confirmed via dissection.

### Videofluoroscopic Swallow Study (VFSS)

Fourteen rats underwent VFSS 2-months post-radiation for objective assessment of the oral and pharyngeal stages of swallowing using a custom, miniature, low-energy fluoroscope (The LabScope, Glenbrook Technologies, Randolph, NJ) [27–30]. Prior to testing, rats underwent behavioral conditioning tasks for several weeks to ensure familiarity of the test environment and food/liquid test items. For VFSS, rats underwent an overnight restriction of either water or food to maximize drinking or eating performance, respectively. During this time, rats had free access to standard enrichment (e.g., wooden block for chewing, PVC pipe for burrowing). Assessment of drinking and eating were performed on separate consecutive days. During testing, individual rats were enclosed within a VFSS test chamber and positioned on a remote-controlled lift in the lateral plane of the fluoroscope. One end-cap on the chamber was fitted with either a drinking or pellet bowl, positioned ~2 mm above the chamber floor. For drinking, the bowl was filled (~1.5 mL) with vanilla flavored thin liquid contrast (74.7% of 30% sucrose in DI water, 0.3% non-alcoholic vanilla extract, and 25% Omnipaque 350; GE Healthcare, Marlborough, MA) via a custom syringe delivery system. For eating, the bowl was filled with 10 peanut butter-flavored barium pellets (40% weight/volume; circular shape, 2.15–2.17 g per pellet; AFB International, St. Charles, MO), which have a dry, crunchy consistency. While rats typically remain stationary at the bowl during drinking, we found that they often take the food pellets to the opposite end of the chamber for eating. Therefore, during chewing trials, a custom adjustable end-cap that reduces the internal length of the chamber by 5 cm increments was used to restrict the rat to a space near the food bowl. The X-ray beam (40 kV, 0.2 mA) was activated during periods when the rat was actively drinking or eating, and AVI recordings were captured at 60 frames/second. To record sufficient data while also limiting radiation exposure, each rat was given multiple brief trials of drinking (up to 3 trials lasting ~1–3 minutes each) and eating (up to 2 trials lasting ~5–10 minutes each) as needed within a 1-hour period.

#### Jaw movement and bolus flow

The fluoroscopy videos were initially viewed in Pinnacle Studio video editing software (Pinnacle Systems, Inc., Mountain View, CA) to identify episodes of uninterrupted drinking and eating. Three non-overlapping 5-second episodes of drinking and 5 episodes of fully consumed pellets were extracted from the raw video for subsequent analysis using a custom VFSS analysis software, JawTrack™. This software performs semi-automated analysis of jaw motion and bolus flow dynamics to calculate several swallowing outcome measures [29,30]. Drinking-based outcome measures include lick rate (i.e., number of licks per second; measured in cycles per second or Hertz, Hz), swallow rate (i.e., number of swallows per second; measured in Hz), inter-swallow interval (i.e., time between sequential swallows; seconds), and pharyngeal transit time (i.e., time between the swallow onset and swallow end frames; measured in milliseconds, ms). Eating-based outcome measures include jaw opening velocity and distance during chewing and the length of time to eat each individual pellet (measured in seconds, s). Jaw opening was analyzed because contraction of the submental muscles facilitates this movement.

#### Tongue protrusion

The maximum protrusion distance (millimeters, mm) of the tip of the tongue during spontaneous drinking for each rat was measured. During the lick cycle, when the jaw was perfectly aligned in the video, TIF images were taken once the bead in the tip of the tongue was clearly visible at the point of maximum tongue protrusion and again during maximum tongue retraction. Using ImageJ, the following 3 landmarks were identified on each image: maxillary first molar, tongue bead, and mandibular first molar. Pythagorean theorem with the molars as stationary landmarks was used to determine the distances between each of the points (mm) and the angles between them.

### Lick force

The maximum voluntary lick force (MVLF) of each rat was analyzed using a custom designed force lickometer system (Med Associates; Fairfax, VT) as previously described [29,31]. The system administers 30% sucrose solution via ball bearing spout. In the chamber, the spout was inserted at a 20° angle through a vertical slit in the end-cap and the platform was height-adjusted to align the rat’s head for optimal tongue contact. Rats underwent two non-consecutive days of behavioral training to prepare animals for the testing condition and environment. During conditioning and testing, the spout force-tension mechanism was set to 2–4 grams (g), which is well below the typical lick force for rats. Tongue contact against the spout during drinking was measured (g) by the lickometer’s force transducer and data was recorded and displayed via LabChart software (ADInstruments; Colorado Springs, Colorado). Animals were monitored in real-time using a webcam (V-u0018, Logitech; Newark, CA) that was synchronized to the force waveform data via LabChart. Force-lickometer testing was performed immediately following VFSS testing while still under water restriction to motivate participation. In order to collect ~2 minutes of active drinking data, each rat was given up to three, 5-minute drinking trials within a 1-hour period.

LabChart files were first analyzed to identify 3–4 of the longest licking bouts, which consists of uninterrupted drinking at the spout lasting >20 seconds. Licking bouts typically totaled ~2–3 minutes. Only lick-force data confirmed as actual licking at the spout in the synchronized webcam videos were included in data analysis. The peak-to-peak amplitude (g) of each lick was calculated in LabChart and exported into a Microsoft Excel file. The 10 highest amplitude values among the collective bouts were averaged to obtain each rat’s MVLF value.

### ELISA

TGFβ1 protein expression was analyzed because it is the most important growth factor controlling collagen formation and its biological activity occurs at the protein level. To analyze if radiation provokes TGFβ1 expression, the mylohyoid muscle was collected, incubated in RNAlater for 48 hours, and then subsequently transferred to -80°C for storage. Tissue samples were homogenized with 11 volumes of buffer containing 10 mM Tris, 10 mM NaCl, 0.1 mM EDTA, 15 mM mercaptoethanol in dH_2_O then centrifuged at 12,000 g for 20 minutes. Supernatants were collected, aliquoted and stored at -80°C until subsequent analysis. Total protein concentrations were quantified using bicinchoninic acid (BCA) protein quantification assay. An ELISA was performed according to the manufacturers protocol (Invitrogen, USA) to measure active TGFβ1 expression in the muscle samples. After thawing the protein samples and centrifuging them at 10,000rpm to remove any precipitate, 20μl of the sample was combined with assay buffer and added to each well in duplicate. Standard curves were used to measure the quantity of each sample. Lysis buffer alone was analyzed to control for non-specific staining. The absorbance was measured by spectrophotometer at 450nm. TGFβ1 levels in the tissue were normalized to the protein concentration from the BCA assay and are expressed as TGFβ1/pg of protein.

### Histology

To assess collagen content following 64Gy radiation and determine if there are differences in the amount of collagen between irradiated muscles, the geniohyoid and anterior digastric muscles were snap frozen in freezing media (OCT compound, Tissue-Tek, CA) using liquid nitrogen-isopentane method and stored at -80°C. Serial cross sections were taken with each muscle at 10μm via cryostat. Tissue specimens were first stained with Hematoxylin and Eosin to visualize general morphologic changes. Additional sections were stained with Picrosirius red using similar methods outlined by Hadi et al [32] for frozen, un-fixed muscle. Slides were air dried, then hydrated by transferring through descending concentrations of ethanol (100%, 80%, and 40%) for 10 seconds each. Slides were then fixed with 4% PFA for 30 minutes. Staining was performed using a 0.1% solution of Sirius red F3B in saturated aqueous solution of picric acid for one hour at room temperature. Sections were then rinsed in 0.5% acetic acid for 2 min, dehydrated in ascending concentrations of ethanol (40%, 80%, and 100%) and subsequently immersed in three stages of Xylene for 10 seconds each. Sections were then covered with resinous mounting medium and glass cover slip.

Stained slides were examined with a Nikon TiE inverted microscope with Nikon Elements Advanced Research software (Nikon Corp, Melville, NY). Two sections (~100 μm apart) taken at the widest part of each muscle were examined for each animal (n = 5 per group). For each section, 5 randomly selected areas were imaged at 20x and analyzed with ImageJ [33]. Collagen was quantified by calculating the fractional area of red staining within each image as previously described [34].

### Immunofluorescence

To assess if fiber type distribution and fiber area were affected at 2-months post radiation, myosin heavy chain (MyHC) isoforms I, IIa, IIb, and IIx were analyzed in the geniohyoid and digastric muscles using methods previously described [35]. These MyHC subtypes were chosen as previous studies have shown that they are distributed in the normal rat submental muscles [36]. Unfixed tissue sections were blocked in 10% normal goat serum for 1 hour and then incubated overnight at 4° with mouse primary antibody cocktails: SC-71 IgG1 for MyHC IIa and BF-F3 IgM for MyHC IIb; SC-71 IgG1 and 6H1 IgM for MyHC IIx; or BA-F8 IgG2b for MyHC I (Developmental Studies Hybridoma Bank). To detect myofiber borders, rabbit anti-laminin (Sigma, L9393) was applied to each section. These antibodies have been extensively used and validated in previous studies [35,37]. Secondary antibody-only control sections were processed concurrently. Sections were then washed three separate times for 5 minutes in PBS and then incubated for an hour in the following fluorescent secondary antibodies: donkey anti-rabbit IgG A350 and goat anti-mouse IgG2b A555, IgG1 A633, IgM A546 (Invitrogen). Sections were then washed and mounted under a coverslip with Vectashield mounting media (Vector Laboratories, CA). Fluorescent images were captured using a Nikon TiE inverted microscope and NIS-Elements AR. Secondary antibody-only controls were used to optimize imaging parameters. Exposure times remained identical across all images. Three sections were analyzed from each rat. For each section, images were taken across multiple regions via semi-automated image acquisition with a 20x objective.

Quantitative analysis of myofibers was performed on six images per animal. The number of images chosen allowed for analysis of >300 myofibers per animal. Semi-automated analysis was conducted using NIH FIJI software with MyoSight plug-in [38]. Laminin staining was used to identify fiber borders (Regions of Interest; ROI). Incorrect ROI were manually corrected. Myosight then determined whether the average pixel brightness for each ROI exceeded the threshold for the designated MyHC signal. Muscle fiber size was measured from each ROI as the minimum Feret diameter (μm^2^). Results were saved via text file and data from each myofiber was averaged to produce one data point for each animal.

### Statistics

All statistical analysis was performed using IBM SPSS software (V28, Chicago, IL). T-tests were used to compare differences in swallowing metrics, profibrotic factors, and muscle fiber type between radiated and sham-treated controls. There was homogeneity of variances for all variables for treatment and controls as assessed by Levene’s test for equality of variances (p >0.05). Therefore, standard independent sample t-tests were performed and p<0.05 was considered statistically significant. To assess relationships between changes in tongue kinematics during swallowing and profibrotic markers in the submental muscles, Pearson’s product moment correlations (r) were calculated comparing TGFβ1 expression, collagen content, and tongue protrusion distance across conditions. Only variables found to be significantly different with t-test were included in correlation analysis.

## Results

### Swallowing—bolus transit during drinking

**Fig 1:**
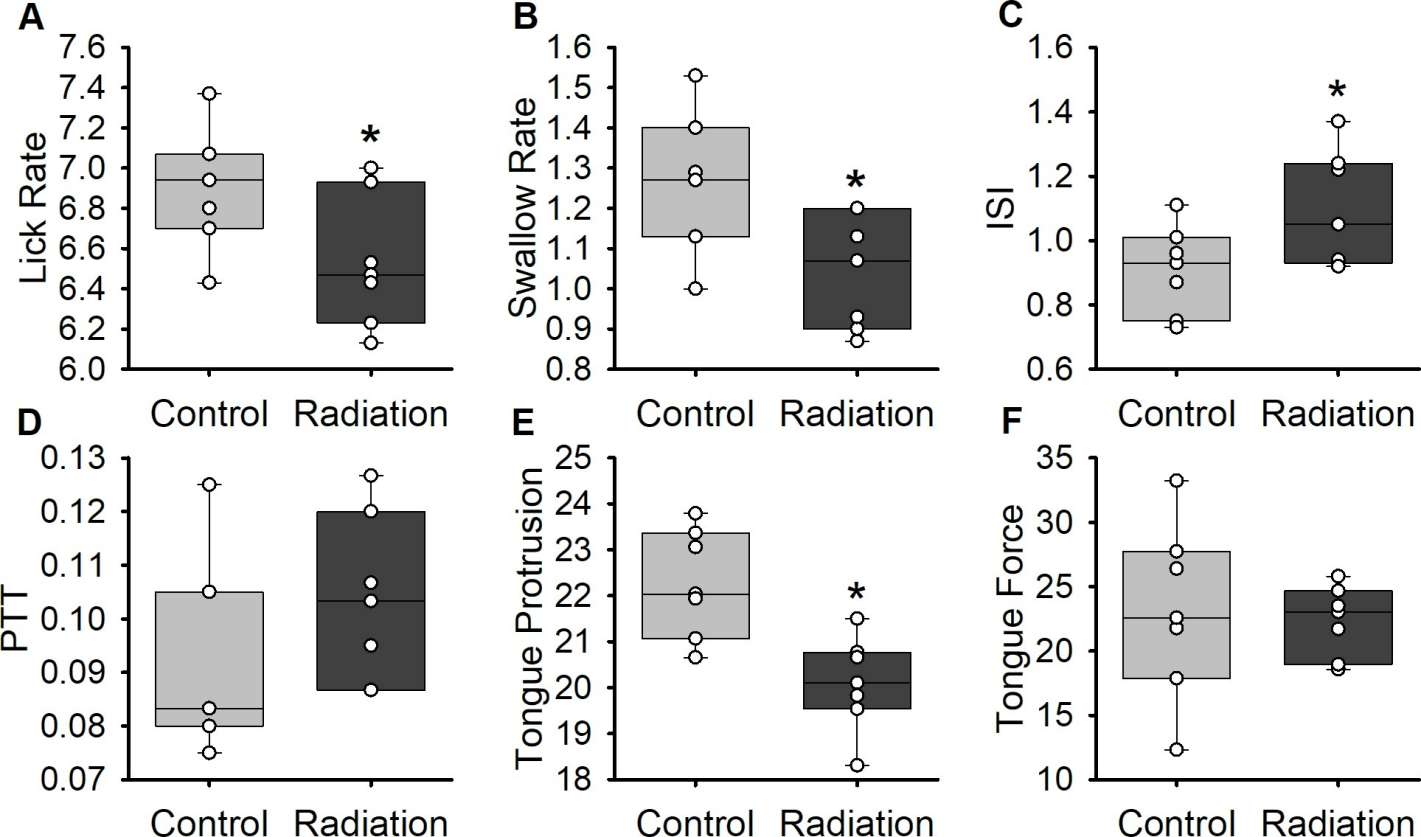
Changes in lick and swallow metrics in rodents 2-months post treatment. Results demonstrate the mean and standard deviations for both groups. (A-E) Lick rate (Hz), swallow rate (Hz), inter-swallow interval (ISI; seconds), pharyngeal transit time (PTT; ms), and tongue protrusion (mm) were obtained from videofluoroscopic swallow studies. (F) The maximum voluntary lick force was obtained from force lickometer. The boundaries of each box depict the interquartile range and the line within the box marks the median. Whiskers above and below the box indicate the 90^th^ and 10^th^ percentiles. Each circle represents the mean value for an individual animal. Statistically significant differences (p < 0.05) between groups are indicated by an asterisk.

To determine the effects of 64Gy radiation on swallowing function at 2-months post treatment, jaw movement and bolus flow were analyzed during drinking. Statistical results for the swallowing variables are summarized in Fig 1. Significant differences between treatments were found for lick rate, swallow rate, and inter-swallow interval (Fig 1A–1C). During drinking, irradiated rats exhibited significant decreases in lick rate (*t*(12) = 2.253, *p* = .044) and swallow rate (*t*(12) = 2.696, *p* = .019) compared to sham-treated rats. There was a significant increase in inter-swallow interval with irradiated animals compared to the sham group (*t*(12) = 2.183, *p* < .05).

### Tongue movement and strength during drinking

To assess alterations in tongue movement during drinking, the distance and force of the protruding tongue were measured during drinking. Irradiated rats exhibited significant decreases in the maximum tongue protrusion distance compared to controls (*t*(12) = 4.601, *p* < .001; Fig 1E). No differences were found between groups with the maximum voluntary lick force (Fig 1F).

### Mastication analysis

**Fig 2:**
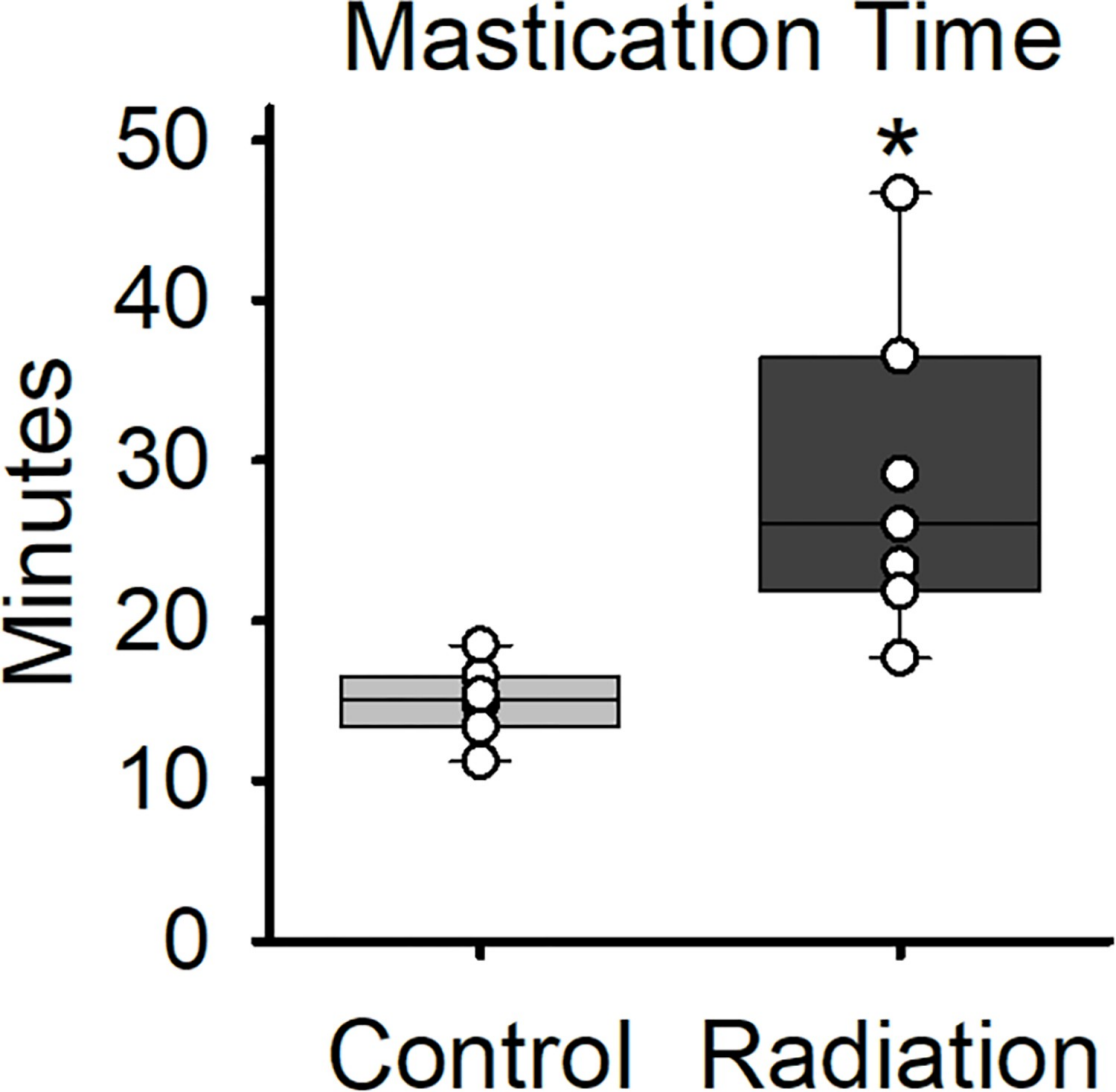
Changes in eating behavior in rodents 2-months post treatment. Results demonstrate the mean and standard deviations for both groups. The mastication time (seconds) was measured by analyzing the length of time it took each rat to eat each individual pellet. The boundaries of each box depict the interquartile range and the line within the box marks the median. Whiskers above and below the box indicate the 90^th^ and 10^th^ percentiles. Each circle represents the mean value for an individual animal. Statistically significant differences (p < 0.05) between groups are indicated by an asterisk.

To determine differences in eating behavior after irradiation, videofluoroscopy was used to monitor animals during self-feeding of barium food pellets. The time it took to consume five pellets was analyzed. Irradiated rats took significantly more time to consume 5 pellets compared to the control group at 2-months post-radiation (t(12) = 3.591, p = 0.01; Fig 2).

### Expression of TGFβ1

**Fig 3:**
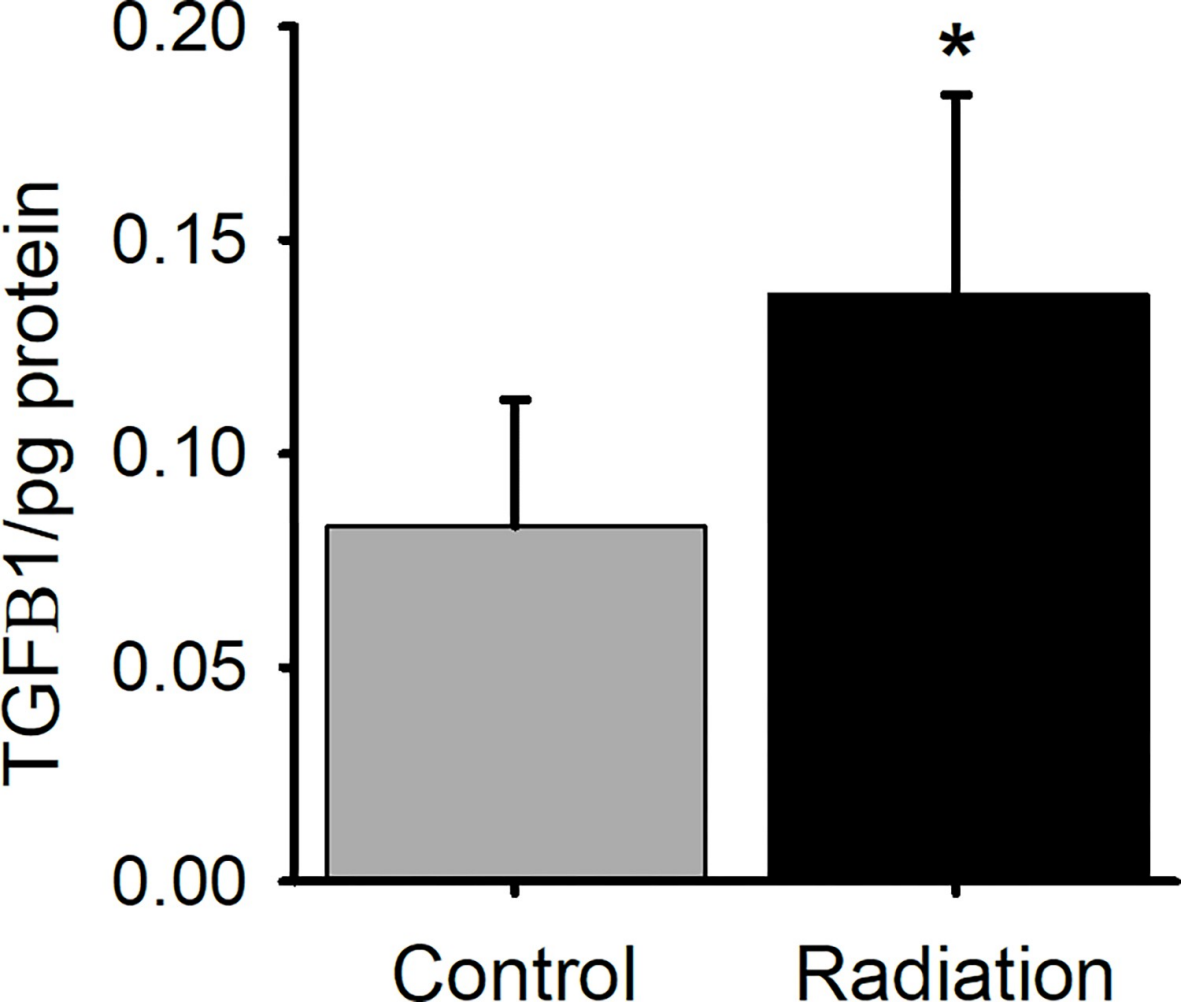
Changes in profibrotic response post radiation. 64Gy of radiation increases TGFβ1 expression in the mylohyoid muscle compared to sham treatment as determined by ELISA. TGFβ1 levels in the tissue were normalized to the protein concentration from the BCA assay and are expressed as TGFβ1/pg of protein. Radiation and sham treated controls were compared by t-test. Statistical significance of p< 0.05 is indicated by an asterisk.

To determine if radiation provokes profibrotic signaling in the mylohyoid muscle two months post treatment, we examined the expression of TGFβ1 using ELISA. TGFβ1 protein expression significantly increased within the irradiated mylohyoid muscle compared to sham-treated muscles (*t*(10) = 2.559, *p* < .037) 2-months following 64Gy of radiation (Fig 3).

### Histological analysis of collagen

**Fig 4:**
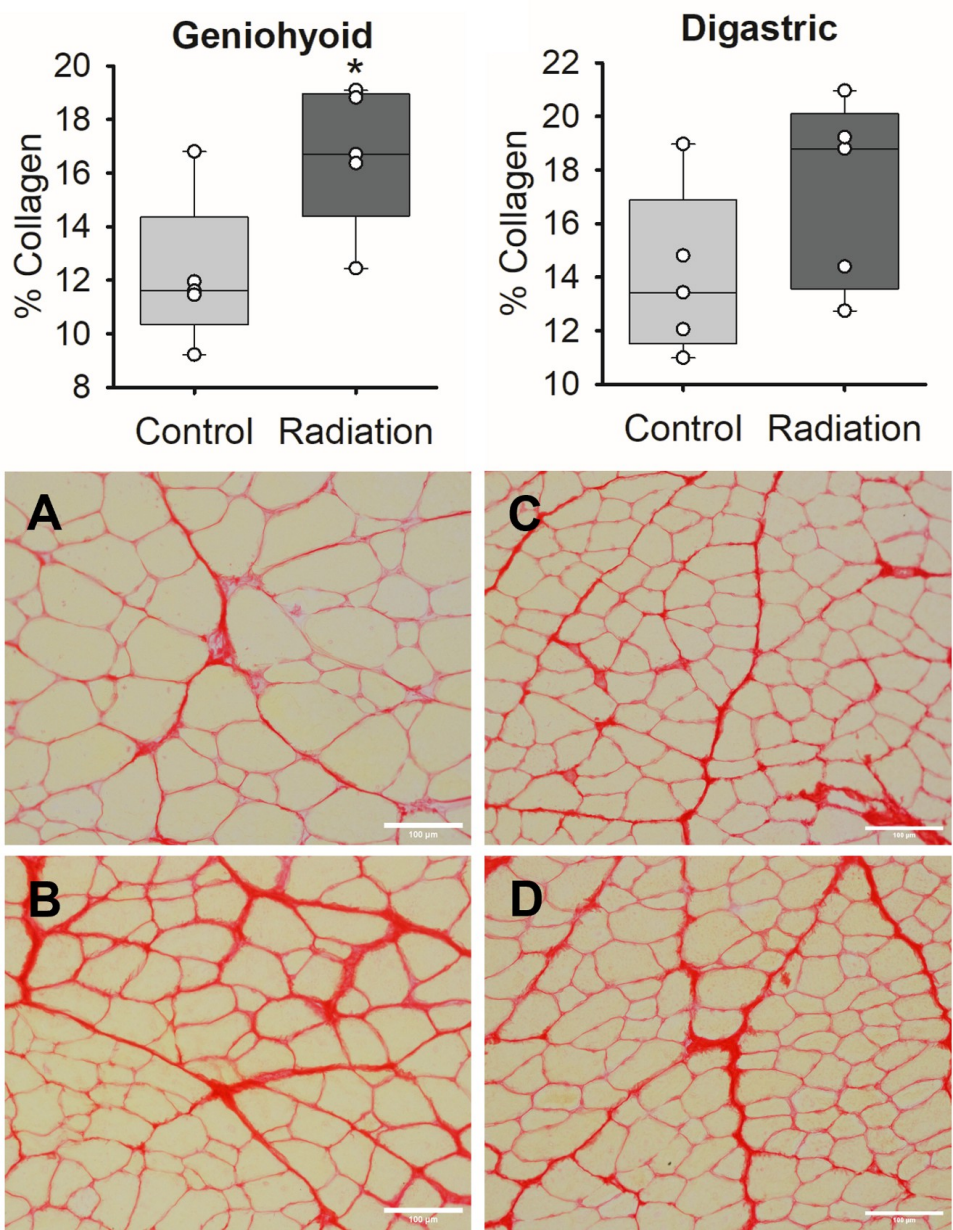
Changes in collagen content post radiation in the geniohyoid and anterior digastric muscles. Significant increases in the % of collagen content were observed post-radiation in geniohyoid muscle compared to sham treatment (p<0.05 is shown as *). The boundaries of each box depict the interquartile range and the line within the box marks the median. Whiskers above and below the box indicate the 90^th^ and 10^th^ percentiles. Each circle represents the mean value for an individual animal. Images show the cross section of the geniohyoid (A & B) and digastric (C & D) muscles from sham (A, C) and radiated (B, D) treated rats. Tissue was stained with Picrosirius red. Images are at 20x (Bar, 100μm). Red staining denotes collagen fibers and yellow indicates muscle bundles.

The percentage of collagen abundance in the geniohyoid muscle significantly increased after radiation (*t*(8) = 2.599, *p* = .032) compared to sham controls. Collagen content in the geniohyoid post-radiation was 16.7%±2.4 compared to 12.2%±2.5 in the sham-treated muscle (Fig 4). No differences were found in the percentage of collagen in the anterior digastric muscle between irradiated (17.2%±3.1) and sham (14.0%±2.8) treatments.

### Myosin heavy chain composition

**Fig 5:**
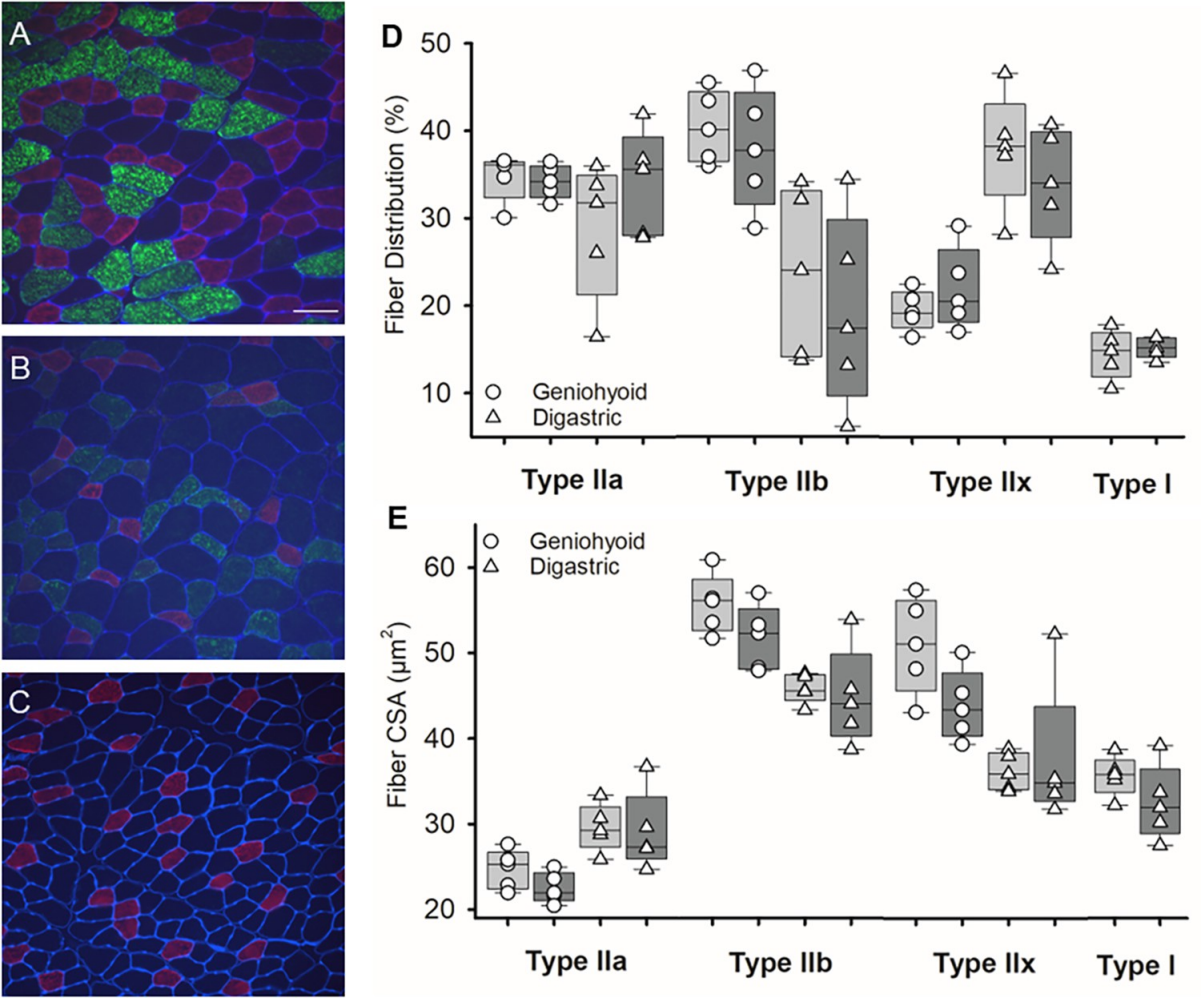
MyHC fiber type composition and size in the geniohyoid and digastric muscles after radiation or sham treatment. **Representative images of MyHC expression in the digastric muscle.** (A) Section was stained for expression of MyHC IIa (red), IIb (green), and laminin (blue). (B) Section was stained for expression of MyHC IIa (red), IIx (green), and laminin (blue). (C) Section was stained for expression of MyHC I (red) and laminin (blue). (D) The percentage of fibers positive for MyHC type IIa, IIb, IIx, and I in the sham (light grey) and radiated (dark grey) treated animals. (E) The fibers cross sectional area (CSA) for each muscle fiber. The boundaries of each box depict the interquartile range and the line within the box marks the median. Whiskers above and below the box indicate the 90^th^ and 10^th^ percentiles. Each circle represents the mean value for an individual animal.

To determine if radiation modulates contractile properties of the submental muscles, the MyHC composition was evaluated by immunofluorescence and microscopy. No differences between groups were found in the percentage of positive MyHC IIa, IIb, and IIx in the geniohyoid or digastric muscles (Fig 5). The majority of geniohyoid myofibers were positive for MyHC IIa and IIb irrespective of treatment groups. Type I MyHC fibers were extremely rare in the geniohyoid muscle of the rat and thus, data was not analyzed. In the digastric, the majority of the muscle fibers were positive for MyHC IIa. No differences between groups were found in fiber size cross-sectional areas (CSA) with geniohyoid or digastric muscles. MyHC IIb and IIx fibers exhibited the largest CSA in both muscles.

### Relationship between profibrotic factors and tongue kinematics

There was a strong negative correlation between the displacement in tongue protrusion during drinking and collagen content in the geniohyoid muscle (r = -0.67) and TGFβ1 expression in the mylohyoid muscle (r = -0.73).

## Discussion

The purpose of this study was to test possible mechanisms for swallowing dysfunction after clinically relevant total doses of radiation to the submental muscles. It was hypothesized that irradiated rats would demonstrate reduced tongue force and displacement during behaviors of deglutition. It was also hypothesized that radiation would provoke profibrotic markers and/or muscle fiber type switching within the irradiated submental muscles, which correlate with changes in tongue movement. Significant decreases in tongue protrusion during drinking and negative changes in licking and swallowing rate were observed. Results also showed that TGFβ1 increased in mylohyoid muscle and collagen content increased significantly in the geniohyoid muscle after radiation. Negative correlations were found between tongue movement and TGFβ1 and total collagen expression. These experiments indicate that irradiating the submental muscles alters lingual movement and that profibrotic phenotype may be in part contributing to swallowing dysfunction post-radiation treatment.

Swallowing function in humans after radiation treatment can result in a complexity of impairments that affect tongue displacement, hyolaryngeal excursion, anterior movement of the pharyngeal wall, as well as other movements involved in swallowing. It is difficult to determine the direct effects of radiation on the swallowing muscles based on clinical data alone. Analyzing animal models where radiation is targeted to one set of swallowing muscles provides opportunities to examine the functional outcomes to swallowing and the underlying cause for impairment. The current study is a continuation from a previous publication where our group examined behavior changes during radiation treatment. From this work, we found that licking and swallowing dysfunction started at the end of radiation treatment to the submental muscles [10]. Similar to our previous findings, we observed decreases in swallow and lick rate and increases in inter-swallow interval in irradiated animals at two months post. Therefore, these swallowing impairments persisted from the last week of radiation treatment to two months post treatment. To further explore the underlying kinematic deficits that may be causing these functional changes, the same rodents were later injected with radiopaque markers in the tongue and underwent videofluoroscopy swallow studies with a customized fluoroscope capable of recording at 60 frames per second (fps). This allowed for more detailed analysis of tongue kinematics during swallowing.

Previous human and animal studies have suggested that radiation can reduce muscle strength and speed. A study by Benedict et al analyzing maximum tongue displacement following radiation of the hyoglossus muscle (7Gy x 5) in an anesthetized rat observed decreases in tongue movement post treatment [8]. Since the submental muscles are indirectly involved in tongue movement and jaw opening [25], we explored the possibility that changes in tongue displacement was a possible cause for the alterations in licking and swallowing that were observed post treatment. Results showed decreases in tongue displacement during drinking two months after radiation. Tongue movement is interlinked to the position of the jaw and hyoid bone [39,40], which the submental muscles control. No changes in jaw opening distance or velocity during drinking were found between groups, suggesting that jaw movement did not influence the changes in tongue displacement. Therefore, the tongue does not need to be the target of radiation to result in changes in its movement. This is particularly interesting because the main strategy to optimize radiation is to reduce the dose to organs at greatest risk for radiation-induced dysphagia [41]. Much of this work focuses on the function of each structure and does not factor in the complex sensorimotor integration that controls the swallowing process. Previous work also demonstrated that radiation can reduce tongue strength. Specifically, in anesthetized model decreases in maximum tongue force and reductions in the speed of tongue muscle contraction were observed after radiation to the tongue muscles (i.e., hyoglossus and genioglossus) [8,11]. We were interested in determining if irradiating submental muscles also effected tongue strength and if it was a potential cause for abnormal changes in licking and swallowing. This information is important clinically, as it may support the use of tongue strength exercises immediately post radiation. Since swallowing requires submaximal tongue strength, we examined voluntary tongue forces with a natural drinking behavior. In contrast to previous studies, our results showed no differences in tongue force during drinking between radiated and control animals. These variations in results may be related to methodological differences between lingual strength measures taken, as measuring maximum tongue force in relative isolation is distinctly different than tongue force used during swallowing. It may also be related to the fact that the tongue was not directly radiated in this study. It would be interesting to determine if maximum lingual strength analysis is a sensitivity measure for early identification of patients susceptible for developing radiation-induced dysphagia.

TGFβ1 is a key mediator of fibrosis, and its expression is one of the most consistent findings following radiation [42]. Increased levels have been observed in numerous irradiated tissue and cells, including human skin and cervical strap muscles [43,44]. King et al demonstrated upregulations in mRNA TGFβ1 and other growth factors in submental muscles 2- and 4-weeks after radiation to this region [9]. Given that TGFβ1 can differ in its activity and level of protein expression, depending on the tissue studied, further analysis of the swallowing muscles was warranted. Our data show that active TGFβ1 protein expression is significantly increased in irradiated mylohyoid muscles at two months post. TGFβ1 is a multifunctional cytokine that plays a role in several biological activities. TGFβ1 signaling is initiated when the protein binds to its serine/threonine kinase receptor on the cell membrane, leading to phosphorylation of SMAD2/3 complex or other signaling pathways (i.e., mitogen activated protein kinase). This in turn activates or represses transcriptional pathways involved in regulating the synthesis of ECM-related genes. Previous cell culture studies have demonstrated that radiation upregulates type I collagen expression through the TGF/Smad3 signaling pathway [45], which may indicate that TGFβ1 signaling is inducing collagen expression in the irradiated swallowing muscles. TGFβ1 also exhibits cellular-mediated changes. For example, activation of TGFβ1 after injury leads to the differentiation of fibroblasts into myofibroblasts, which produce excessive ECM resulting in fibrosis. Inhibiting this cytokine by blocking its receptors/SMAD-dependent signaling or oxidative stress properties is one possible approach to downregulate the fibrotic response post-radiation. Given that TGFβ1 is known to regulate a broad range of biological processes, further gene sequencing studies are warranted to analyze differentially expressed genes at different time points in the swallowing muscles post-radiation.

Clinical evidence indicates that total radiation dose-volume parameters are predictive of late onset dysphagia. Specifically, the volume of the submental muscles receiving >60Gy are associated with greater swallowing toxicity [21,23,46,47]. Therefore, we tested the possibility that clinically relevant radiation dose volumes provoke more substantial muscle damage, which in turn results in increases in collagen in the irradiated muscle. We observed ~4% increase in collagen in the geniohyoid muscle two months after 64Gy of radiation, which was significantly different compared to the sham-treated group. In contrast, previous animal studies utilizing lower fractionated dose schemes (22Gy or 35Gy) showed no measurable changes in total collagen in the irradiated swallowing muscles at two weeks or five months post treatment [8,11]. While different collagen stains were used across studies (Picrosirius red versus Masson trichrome), these staining techniques have been shown to be comparable [48]. If we assume that all swallowing muscles respond similarly to radiation, then comparisons between the aforementioned studies and the current data suggest there are dose-related fluctuations in total collagen accumulation in the swallowing muscles. However, certain muscles may respond differently to radiation. To determine if there are differences in total collagen between the submental muscles, we analyzed collagen content in the anterior digastric muscle following radiation. In contrast to the geniohyoid muscle, we observed no measurable changes in total collagen in the irradiated digastric muscle. In a previous study, alterations in the inflammatory cytokine response were observed in the mylohyoid and geniohyoid muscles post radiation, but minimal to no changes were seen in the irradiated anterior digastric muscle [9]. We have also previously shown with a simulated depth-dose curve that the anterior digastric muscle is receiving about the same dose per fraction compared to the geniohyoid muscle [7]. Therefore, the anterior digastric muscle appears to be less responsive to radiation. This further indicates that radiation induced changes in collagen are tissue specific. It is possible that the increases in collagen in the geniohyoid muscle represents a transient effect as part of the remodeling phase of the wound healing process. Later time points (i.e., >9-months) are needed to determine if collagen is a permanent aberrancy (fibrosis) in this muscle. Further investigations are also warranted to determine if fibrotic inhibitors are reducing the synthesis or breaking down of collagen at the protein level, which may explain the modest increase in the amount of collagen.

We also tested the possibility that muscle fiber type composition and size in the geniohyoid and digastric muscles was an underlying mechanism of swallowing dysfunction post radiation. The distribution of fiber types in geniohyoid and digastric muscles were similar to previous histological studies in normal tissue [36]. The geniohyoid muscle had greatest percentage of type IIb fibers or fast glycolytic fibers, representing ~40% in controls and ~38% in radiated tissue. Digastric muscle fibers exhibited the greatest percentage of fast oxidative fibers type IIa and fast glycolytic fibers type IIx. Slow contracting muscle fibers, MyHC type I, were only observed in the digastric muscle. Our findings showed no difference in MyHC isoform profiles in radiated muscles compared to untreated controls. Therefore, changes in fiber type composition in submental muscles are not likely the cause for early changes in lingual function seen in irradiated rodents. These findings are not consistent with previous studies examining radiation to the hindleg or laryngeal muscles. Kim et al examined gene expression of MyHC isotypes in laryngeal muscles and found gradual decreases in MyHC isotypes IIa, IIb, and IIx starting 3 days after a single radiation doses >10Gy [20]. Hardee et al analyzed post radiation changes to myofiber size with a single dose of 16Gy to hindlimb [19]. Their findings demonstrated that 16Gy resulted in smaller diameters of type IIa and IIb myofibers. It is possible that previous changes in muscle fiber type composition and size may not be consistent with the lower fractionated doses used in current study.

There are limitations to our study that warrant further discussion. Experiments were performed in adult rats and therefore, the effects of aging cannot be determined. There was an unplanned break in radiation treatment, which likely resulted in greater acute side effects to mucosal tissue. Thirdly, due to the small size of each muscle, molecular and protein assays were performed in different submental muscles; however, all muscles tested were within the field of radiation. Lastly, although VFSS data were collected using an increased frame rate (60 fps), analyzing jaw movement and tracing the radiopaque bead in the tongue during eating was challenging due to motion artifact and therefore data could not be collected. Machine-learning efforts are underway to overcome this limitation in our future studies.

In conclusion, we show that decreases in tongue displacement may be one of the kinematic causes for alterations in licking and bolus flow during swallowing post radiation. We also provide evidence that there is a profibrotic transition within the irradiated swallowing muscles early after radiation treatment, which correlates with alterations in swallowing function. Increases in collagen content and TGFβ1 expression were observed post-radiation. The observed changes may represent adaptations of the ECM associated with the wound healing process following radiation to the submental muscles.
